# Genome-Wide Association Study of Exercise-Induced Fat Loss Efficiency

**DOI:** 10.3390/genes13111975

**Published:** 2022-10-29

**Authors:** Aleksandra Bojarczuk, Eugenia A. Boulygina, Magdalena Dzitkowska-Zabielska, Beata Łubkowska, Agata Leońska-Duniec, Emiliya S. Egorova, Ekaterina A. Semenova, Liliya B. Andryushchenko, Andrey K. Larin, Edward V. Generozov, Pawel Cięszczyk, Ildus I. Ahmetov

**Affiliations:** 1 Faculty of Physical Culture, Gdansk University of Physical Education and Sport, 80-336 Gdansk, Poland; 2 “Omics Technologies” OpenLab, Kazan Federal University, 420021 Kazan, Russia; 3 Laboratory of Genetics of Aging and Longevity, Kazan State Medical University, 420012 Kazan, Russia; 4 Department of Molecular Biology and Genetics, Federal Research and Clinical Center of Physical-Chemical Medicine of Federal Medical Biological Agency, 119435 Moscow, Russia; 5 Research Institute of Physical Culture and Sport, Volga Region State University of Physical Culture, Sport and Tourism, 420138 Kazan, Russia; 6 Department of Physical Education, Plekhanov Russian University of Economics, 115093 Moscow, Russia; 7 Research Institute for Sport and Exercise Sciences, Liverpool John Moores University, Liverpool L3 5AF, UK

**Keywords:** GWAS, polymorphism, genotype, obesity, overweight, training, performance, athletes

## Abstract

There is a wide range of individual variability in the change of body weight in response to exercise, and this variability partly depends on genetic factors. The study aimed to determine DNA polymorphisms associated with fat loss efficiency in untrained women with normal weight in response to a 12-week aerobic training program using the GWAS approach, followed by a cross-sectional study in athletes. The study involved 126 untrained young Polish women (age 21.4 ± 1.7 years; body mass index (BMI): 21.7 (2.4) kg/m^2^) and 550 Russian athletes (229 women, age 23.0 ± 4.1; 321 men, age 23.9 ± 4.7). We identified one genome-wide significant polymorphism (rs116143768) located in the *ACSL1* gene (acyl-CoA synthetase long-chain family member 1, implicated in fatty acid oxidation), with a rare T allele associated with higher fat loss efficiency in Polish women (fat mass decrease: CC genotype (*n* = 122) −3.8%; CT genotype (*n* = 4) −31.4%; *p* = 1.18 × 10^−9^). Furthermore, male athletes with the T allele (*n* = 7) had significantly lower BMI (22.1 (3.1) vs. 25.3 (4.2) kg/m^2^, *p* = 0.046) than subjects with the CC genotype (*n* = 314). In conclusion, we have shown that the rs116143768 T allele of the *ACSL1* gene is associated with higher fat loss efficiency in response to aerobic training in untrained women and lower BMI in physically active men.

## 1. Introduction

The prevalence of obesity has tripled over the past four decades. If these rates do not slow down, it is expected that 2.7 billion adults will be overweight and over 1 billion will be obese by 2025 [1]. In this light, obesity is a significant burden on human health.

One could ask whether we are destined to be obese. Crucially, genetics contribute to the development of obesity. Most obesity cases are polygenic. These are due to the combined net effect of polygenic variants [2]. However, some extreme obesity cases can be developed due to rare mutations (i.e., monogenic obesity) [3]. Both categories of obesity demonstrate inheritance patterns. Heritability is frequently cited as an estimate of the upper boundary for total genetic contributions to a trait [4]. The heritability of obesity ranges between 31% and 90%, depending on the phenotype studied [5]. Further, body mass index (BMI) is demonstrated as a transmissible property shown during childhood [6] and extending into adulthood [7], with an increased degree of heritability in men compared to women and in younger adults compared to the elderly [8]. Recent data suggest that the heritability of BMI is between 40% and 70%. For the waist-to-hip ratio (WHR), the estimates are 30–60% in a population of women twins [9]. The percentage of genetic influence for waist circumference (WC) is reported at 46% in males and 66% in females [10]. The heritability of percentage fat mass, whole-body fat mass, and whole-body lean mass (fat-free mass) are all highly heritable after adjusting for age, sex, ethnicity, height, diabetes status, smoking, dietary intake, and physical activity. Thus, it should be highlighted that when estimating heritability, it is important to acknowledge that there always will be factors accounting for its value, e.g., geographical coordinates, sex, population-specific or ethnic-group effects.

The processes of screening whole genomes and identifying the genetic factors underlying the variation in body composition and another exercise- and health-related phenotypes are designed to ultimately improve strategies for the prevention and treatment of obesity [11]. Genome-wide association studies (GWAS) are one approach, wherein the association between genetic variants and phenotypes is studied [12]. This method is hypothesis-free and seeks to identify single-nucleotide polymorphisms (SNPs) at a much higher resolution than is possible for genome-wide linkage studies and is thus better able to narrow down the associated locus [3]. The first GWAS for obesity traits identified a cluster of common variants in the first intron of the fat mass and obesity-associated (*FTO*) locus that was convincingly associated with BMI [12]. Up until two years ago, nearly 60 GWAS have identified more than 1100 independent loci associated with a range of obesity traits [13].

More recent attention has focused on GWAS and whole-genome sequencing in the aspect of sport and training [14,15,16,17]. Physical activity is associated with a reduced risk of multiple non-communicable diseases [18] and is crucial in the prevention of becoming overweight/obese. Individuals with the same genotype respond more similarly to training than those with different genotypes, indicating that genes play an important role in the determination of individual differences in response to training [19,20]. Moreover, the effects of exercise differ greatly among individuals, depending on lifestyle factors and genetic backgrounds [21]. A large and growing body of literature has investigated gene variants and their association with responses to aerobic exercise. For instance, a bioinformatics analysis study conducted by Ghosh et al. found that the greatest number of SNPs were annotated to the peroxisome proliferator-activated receptor (PPAR) signaling pathway, suggesting its importance in aerobic trainability [14].

Recent developments in gene variants and their association with responses to aerobic exercise have been extended to gene variants and exercise-induced fat loss. Several researchers have reported the importance of the following genes and their polymorphisms in fat loss: *FTO*, melanocortin 4 receptor (*MC4R*), peroxisome proliferator-activated receptor gamma (*PPARG*), peroxisome-proliferator-activated receptor delta (*PPARD*), PPARG coactivator 1 alpha (*PPARGC1A*), leptin (*LEP*), leptin receptor (*LEPR*), adiponectin, C1Q and collagen-domain-containing (*ADIPOQ*), adrenoceptor beta 2 (*ADRB2*), adrenoceptor beta 3 (*ADRB3*), insulin induced gene 2 (*INSIG2*), and fatty-acid-binding protein 2 (*FABP2*) (comprehensively reviewed in [22]). These data also show that the effectiveness of physical exercise on fat loss and improvement of aerobic capacity varies considerably between individuals [11,23,24]. This also means that shedding fat and changes in obesity-related traits in response to training programs may be more effective for some genotypes than others.

One might suggest that other genetic variants associated with fat loss efficiency in untrained individuals exist. Therefore, the present study aimed to determine DNA polymorphisms associated with fat loss efficiency in untrained young Polish women with normal weight in response to a 12-week aerobic training program using the GWAS approach, followed by a cross-sectional study in athletes.

It was considered that GWAS measures in this group would usefully reflect the genetic variants because the changes in any traits are always greater in untrained subjects as compared to trained ones. The BMI criteria for selecting the subjects reflected the need to become fitter. Such an approach was designed with the promise of revealing new genes and their variants that point to new biology and pathways in exercise-induced fat loss.

## 2. Materials and Methods

### 2.1. Ethics Statement

The Ethics Committee of the Regional Medical Chamber in Szczecin (approval numbers 09/KB/IV/2011 and 01/KB/VI/2017) and the Ethics Committee of Federal Research and Clinical Center of Physical-Chemical Medicine of the Federal Medical and Biological Agency of Russia (approval number 2017/04) approved the protocols for the experiments. The studies were conducted according to the guidelines of the Declaration of Helsinki and Strengthening The Reporting of Genetic Association Studies (STREGA): An extension of the STROBE Statement recommendations [25].

### 2.2. Participants

#### 2.2.1. Polish Women

This study involved 126 Polish European Caucasian women, aged 21.4 ± 1.7 years (range 19–24) with normal weight (i.e., BMI < 25.0 kg/m^2^) and height (167.6 ± 5.7 cm). The women were also involved in the study with a larger sample size [11]/ and therefore previously published participants’ description partly matches the current description (Table 1). However, eligibility criteria required individuals to have their DNA samples genotyped successfully using microarray analysis. In our previous study [11], 163 DNA samples were genotyped for a limited number of SNPs using real-time PCR. By contrast, the 126 were successfully genotyped using the microarray approach. Thus, the remaining 37 samples were rejected from the current study, and the microarray data alone was considered.

None of these women had engaged in regular physical activity in the previous six months. None of the volunteers had any history of musculoskeletal injuries or metabolic and cardiovascular disorders. Participants were non-smokers and abstained from taking medications or supplements known to affect metabolism. At the initiation phase of the study, before the training phase, participants entered a dietary program, and on the basis of individual dietary plans, they were asked to balance their diet to approximately 2000 kcal per day. The participants were requested to keep track of their food intake daily. Food consumption was consulted weekly. The quality and quantity of meals were analyzed and, if necessary, minor improvements were made.

#### 2.2.2. Russian Athletes

This part of the study involved 550 international-level Russian athletes (229 women, age 23.0 ± 4.1 years, height 172.3 ± 8.8 cm; 321 men, age 23.9 ± 4.7 years, height 184.7 ± 10.7 cm) from the following sporting disciplines: alpine skiing (*n* = 11), badminton (*n* = 13), baseball (*n* = 2), basketball (*n* = 52), boxing (*n* = 51), climbing (*n* = 3), cycling (*n* = 19), decathlon (*n* = 1), figure skating (*n* = 8), football (*n* = 22), handball (*n* = 13), heptathlon (*n* = 1), ice hockey (*n* = 20), jumping events (*n* = 12), kayaking (*n* = 31), pentathlon (*n* = 2), powerlifting (*n* = 17), rowing (*n* = 24), rugby (*n* = 40), running 100–400 m (*n* = 17), sailing (*n* = 3), short track (*n* = 4), skeleton (*n* = 3), speed skating (*n* = 25), swimming (*n* = 19), table tennis (*n* = 2), taekwondo (*n* = 8), throwing events (*n* = 9), volleyball (*n* = 30), water polo (*n* = 16), weightlifting (*n* = 25), and wrestling (*n* = 47). The athletes were all Caucasians of Eastern European descent who had never tested positive for doping.

### 2.3. Physical Exercise Training Protocol

Novice participants were first subjected to a week-long familiarization protocol. Within the familiarization, the examined women exercised 3 times a week for 30 min, at an intensity of about 50% of their maximum heart rate (HRmax) [26]. Following this procedure, the main training started. Preceding the main aerobic routine (43 min), a warm-up (10 min) was conducted. A cool-down (stretching and breathing for 7 min) was performed after the workout. The aerobic protocol consisted of two alternating styles of low and high impact. Music tempo was introduced into both styles. A 12-week program of low–high-impact aerobics was divided as follows: (i) 3 weeks (9 training units), 60 min each, at about 50–60% of HRmax, tempo 135–140 beats per minute (BPM); (ii) 3 weeks (9 training units), 60 min each, at 60–70% of HRmax, tempo 140–152 BPM; (iii) 3 weeks (9 training units), 60 min with the intensity of 65–75% of HRmax, tempo 145–158 BPM; and (iv) 3 weeks (9 training units), 60 min with an intensity of 65–80% of HRmax, tempo 145–160 BPM. The same sports instructor provided all 36 training units.

### 2.4. Body Composition Measurements

Anthropometric measurements and bioelectrical impedance analysis were performed. In all participants, body mass and body composition variables before and after the 12-week training program were assessed. Body composition measurements were performed in a fasting state, which lasted at least 8 h. Body mass and body composition were determined using bioelectrical impedance (Tanita TBF 300M electronic scale, Arlington Heights, Illinois, USA). These variables included total body mass (kg), fat-free mass (FFM, kg), fat mass (FM, kg), fat mass percentage (FM%), and BMI (kg/m^2^).

### 2.5. Genetic Approach

This study used a hypothesis-free, genome-wide association study (GWAS) to find gene variants statistically associated with fat loss efficiency. In Polish women, the GWAS method using a custom DNAFit chip was described in [15]. Saliva samples were collected through sterile and self-administered buccal swabs from all women. GWAS was performed externally by AKESOgen, Inc. (Peachtree Corners, GA, USA), that extracted DNA using Qiagen chemistry on an automated Kingfisher FLEX instrument (Thermo Fisher Scientific, Waltham, MA, USA), according to the manufacturer’s instructions and standard operating procedures. DNA content was assessed by PicoGreen and Nanodrop assays. Input to the custom testing array occurred at 200 ng in 20 mL. Amplification, fragmentation, and resuspension were performed using Biomek FXP following Affymetrix’s high-throughput protocol for Axiom 2.0. Hybridization was performed for 24 h at 48° C in a Binder oven, and staining and scanning of the arrays (>600,000 SNPs; DNAFit’s custom microchips) were performed using GeneTitan instrumentation (Thermo Fisher Scientific), all following the same Affymetrix high-throughput Axiom 2.0 protocol. Data analysis was then performed using a raw CEL file data input into the Affymetrix Axiom Analysis Suite (Affymetrix, Santa Clara, CA, USA).

In Russian athletes, molecular genetic analysis was performed with DNA samples obtained from leukocytes (venous blood). DNA extraction and purification were performed using a commercial kit according to the manufacturer’s instructions (Technoclon, Moscow, Russia). Genotyping of the most significant SNP (rs116143768) discovered in the first stage of our study was performed using microarray technology, as previously described [27].

### 2.6. Statistical Analyses

Statistical analyses were conducted using PLINK v1.90, R (version 3.4.3), and GraphPad InStat (GraphPad Software, Inc., La Jolla, CA, USA) software. All SNPs that did not pass the Hardy–Weinberg equilibrium test, genotyping rate, and minor allele frequency (MAF) thresholds (options: MAF 0.01, genotyping rate 0.05, HWE 0.00001 in PLINK) were excluded from further analysis. Paired t-tests were used to detect the significance of dynamic changes. Differences in phenotypes between groups were analyzed using regression analysis adjusted for age and height. Body composition dynamics were calculated by the percentage change of body composition parameters (percentage change from baseline). Spearman’s (non-parametric) correlations were used to assess the relationships between the phenotypes and the *ACSL1* genotypes (dummy coded as 1 and 2 for CC and CT, respectively). The squared correlation coefficient R^2^ was used as a measure of explained variance. Within the GWAS aspect of this study, *p* < 5 × 10^−8^ was set as the statistical threshold (using Bonferroni correction).

## 3. Results

### 3.1. Changes in Anthropometric Measurements

Selected body composition measurements of 126 women in response to a 12-week aerobic training program are presented in Table 1. A total of 73.8% of participants were able to lose their fat mass (i.e., negative changes were observed) in response to a 12-week aerobic training program. On average, participants lost 0.85 kg (range: +7.7 to −7.8 kg) of their FM, 1.19% (range: +6.2 to −9.5%) of their FM%, and 0.61 kg (range: +4.0 to −7.1 kg) of their body mass. On the other hand, participants’ FFM increased (average: 0.34 kg, range: +7.1 to −2.1 kg). Furthermore, changes in BMI and fat mass did not depend on the age, height, baseline body weight, baseline BMI, and baseline fat mass of participants (*p* > 0.05).

### 3.2. Genome-Wide Significant Markers Associated with Fat Loss Efficiency in the Polish Cohort

**Figure 1:**
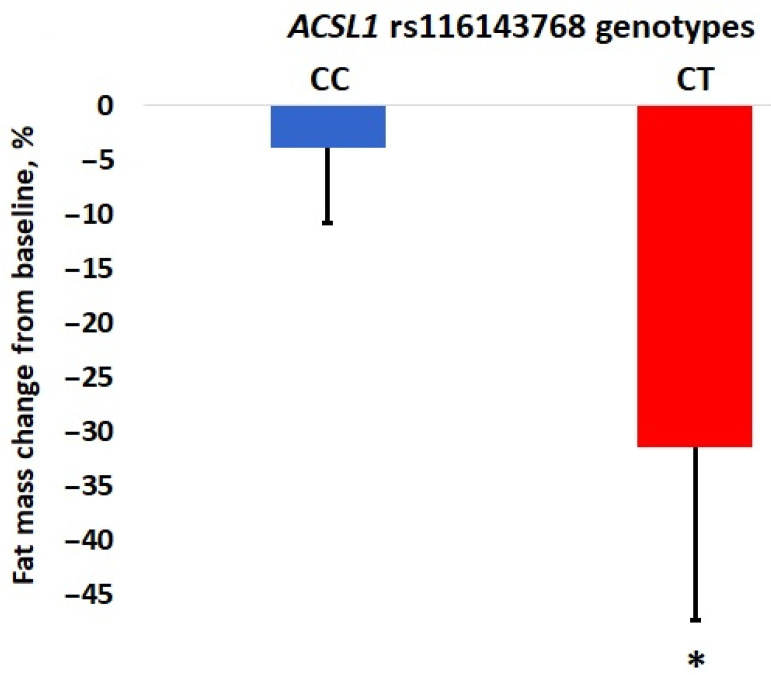
Differences in fat mass changes (%) in response to training between carriers of *ACSL1* rs116143768 CC and CT genotypes among Polish untrained women. * *p* = 1.18 × 10^−9^.

Genotyping of > 600,000 SNPs that passed quality control measures (see Section 2) revealed that of those, only one SNP was identified as significant in the genome-wide context. This was rs116143768, located in the long-chain acyl-CoA synthetase (*ACSL1*) gene (Table 2) with a rare T allele associated with the greatest fat loss efficiency. More specifically, carriers of the *ACSL1* rs116143768 CT genotype (*n* = 4) exhibited a significant decrease in fat mass (change from baseline: −31.4 (16.2) vs. −3.8 (7.9)%; *p* = 1.18 × 10^−9^) as compared to CC genotype (*n* = 122) (Figure 1). The *ACSL1* genotype explained 7.0% of the variation in fat mass changes in response to training. The other four polymorphisms with suggestive genome-wide significance thresholds (*p* < 1 × 10^−6^) are also shown in Table 2. Furthermore, we found that fat-mass-decreasing alleles were associated with positive changes in FFM (Table 2).

### 3.3. Study Involving Russian Athletes

**Figure 2:**
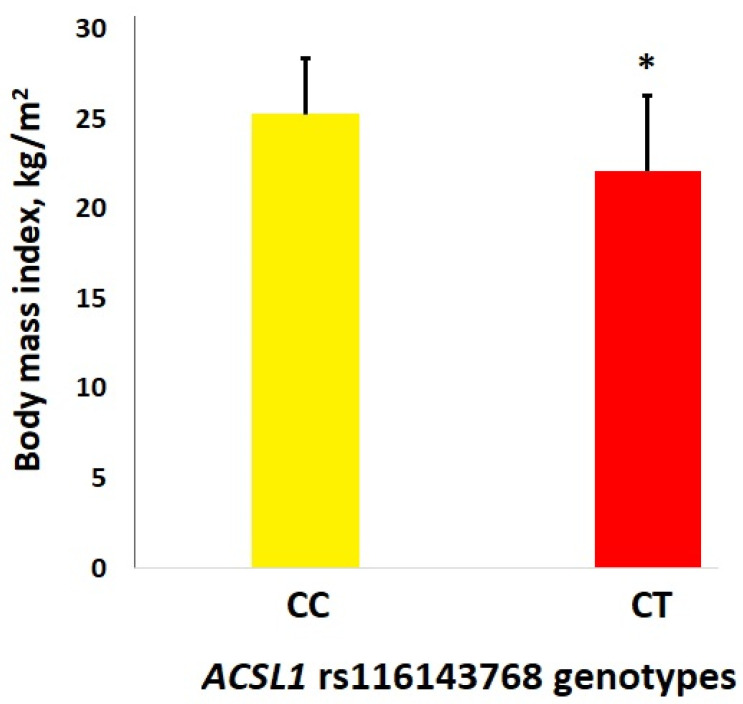
Differences in BMI (kg/m^2^) between carriers of *ACSL1* rs116143768 CC and CT genotypes among Russian athletes. * *p* = 0.046 (adj. for age and height).

Male athletes with the *ACSL1* rs116143768 T allele (*n* = 7) had significantly lower BMI (22.1 (3.1) vs. 25.3 (4.2) kg/m^2^, *p* = 0.046 adjusted for age and height) than subjects with the CC genotype (*n* = 314) (Figure 2). Female athletes with the T allele (*n* = 4) also had lower BMI (21.1 (1.5) vs. 22.1 (3.2) kg/m^2^) than subjects with the CC genotype (*n* = 225), but the difference was not significant (*p* = 0.634 adjusted for age and height).

## 4. Discussion

To our knowledge, this is the first study investigating exercise-induced fat loss using the GWAS approach. The present study was designed to discover new genetic variants associated with the efficacy of weight loss in women whose BMI, according to the WHO criteria, was normal (i.e., ≤25.0 kg/m^2^). Our GWAS also set out to test whether these new SNPs can modulate fat loss expressed as a reduction in fat mass values following 12 weeks of aerobic exercise training. The current study found only one polymorphism, rs116143768 (T/C), within the *ACSL1* gene to be associated with fat loss efficiency after correction for multiple testing, with T allele carriers showing the highest reduction in body fat mass (8.3× fold; *p* = 1.18 × 10^−9^) compared to non-carriers (CC genotype) in the Polish cohort (*n* = 126). Consistent with this finding, we also found that physically active men with the T allele had significantly lower BMI compared to carriers of the CC genotype. This is a novel finding given that the knowledge of the association between SNPs and fat loss achieved by training is limited. 

By contrast, a considerable amount of literature has been published on fat loss (reduction in BMI or fat mass or fat percentage), underlining the role of training and/or diet [28,29,30,31], fat mass, and less preferably using medicines. This is because medications cannot replace physical activity or healthy eating habits as a way to lose weight [32]. Notwithstanding those claims, recent studies have revealed that genetic variants are also associated with weight loss efficiency by means of training and/or diet. For example, SNPs in the *MC4R*, i.e., rs571312 and rs17782313, were significantly associated with a higher reduction in body weight and BMI. The evidence was found in a pooled analysis of 576 individuals with overweight and obesity who were enrolled in two different 12-month weight loss intervention programs. Both of them promoted a balanced diet and physical activity. Therefore, the association of the minor risk allele in the *MC4R* gene (A risk allele in rs571312 and C risk allele in rs17782313) with a higher weight loss and higher BMI loss presented a combined effect of dieting and training. Interestingly, this experiment did not detect any evidence for the SNPs to be associated with fat mass reduction [33]. Many other publications highlight that diets themselves are beneficial for weight loss in individuals possessing certain genotypes [34,35,36].

However, the genetic contribution to efficient weight loss in response to training intervention only remains largely unknown. Nonetheless, it has been demonstrated that, e.g., homozygous carriers of the risk A allele of the *FTO* SNP rs8050136 lose significantly more weight than the C allele carriers. This was shown in 234 Caucasian women when exposed to 6-month moderate or intense exercise [37]. Zarebska et al. demonstrated that CC genotype carriers of *PPARG* rs1801282 polymorphism had a greater decrease in body weight and fat mass compared to the risk allele carriers (G) after a 12-week exercise training program in 201 obese Polish women. The SNP was associated with, e.g., body mass, BMI, fat mass, and fat mass percentage [38]. This is of particular interest since our study identified a genome-wide significant marker (*ACSL1*) rs116143768 associated with fat loss efficiency in response to the 12-week aerobic training program. This is even more significant as the *ACSL1* gene has not been described in the context of fat loss efficacy. 

In mammals, long-chain acyl-CoA synthetase (ACSL) catalyzes the ligation of fatty acids (FAs) to CoA [39]. Thereby, it enhances the transport of FA across the plasma membrane and supplies substrates for most downstream pathways that metabolize FAs [22,40]. Five ASCLs isoforms exist [39,41], each being the product of a separate gene, activating long-chain fatty acids to form acyl-CoAs [42], and each being different in terms of its FA chain length preference, tissue distribution, and subcellular location. These features decide on FAs disparate metabolic fates [43,44]. In rats, *Acsl1* is highly expressed in tissues that undergo high rates of metabolism, including muscle, adipose tissue, cardiomyocytes, and liver cells [45,46]. Thus, it might have a function in aerobic metabolism at the level of the aforementioned cells with highly oxidative function [47], especially since one SNP in *ACSL1*, namely, rs6552828, explained 6.1% of the variance in VO_2max_ changes following aerobic training in sedentary Caucasians [48]. These results do not link with the findings of Yvert et al., who found no association between *ACSL1* rs6552828 with elite endurance athletic status in Caucasians [49]. 

To our knowledge, there is no functional data on the *ACSL1* rs116143768, and this is the first research demonstrating an association of this SNP with fat loss. Thus, it is hypothesized in our study that rs116143768 had a function in the control of adipose tissue metabolism. Thus, it is believed in the current study that the rs116143768 models post-exercise response in the context of body composition. There were significant main effects of training: for body mass, BMI, FM, and FM%. As compared to CC genotype −3.8%, carriers of the *ACSL1* rs116143768 CT genotype showed a significant decrease in fat mass of −31.4%, *p* = 1.18 × 10^−9^. In addition, subjects with the CC genotype had a significantly higher BMI than male athletes with the T allele (25.3 (4.2) kg/m^2^ vs. 22.1 (3.1) kg/m^2^, *p* = 0.046). 

In recent years, there has been an increasing interest in the function of the *ACSL1*. In undifferentiated, proliferating adipocytes, *ACSL1* transcripts were not present. Nevertheless, they were dramatically increased during differentiation, which implies an anabolic role [50]. In contrast, previous research in tissue-specific knockout animal models has also demonstrated its catabolic function in adipocytes [51], the liver [42], and skeletal muscle [52]. These contradictory results imply that the function of the ACSLs might vary depending on the tissue [46]. Although there is insufficient evidence in this current study, i.e., the study is limited by the lack of information on whether the T allele increases or decreases *ACSL1* expression, it is plausible to speculate that it serves a role in the catabolic pathway when combined with physical activity. 

In support of this, according to the transcriptional study of Bolotta et al., the expression of the *ACSL1* gene in vastus lateralis demonstrated a onefold increase in the comparison of life-long high-level training practice athletes versus sedentary subjects in response to exercise [53]. Similarly, acute endurance exercise significantly increased skeletal muscle *ACSL1* gene expression in Caucasian women [54]. Expression of *ACSL1* was also increased in overweight non-trained men who underwent two months of training, including three 60-minute cycling sessions per week at 50% VO_2max_. Although the *ACSL1* mRNA levels were not significantly different after training completion, the fold change in *ACSL1* strongly and positively correlated with the fold change in both fasting and post-prandial total fat oxidation [55]. The authors claimed that these results were in agreement with in vitro findings from [56]. The latter described that ACSL1 indirectly contributes to FA uptake through metabolic trapping [56]. In addition, the authors of [55] referred to the HERITAGE family study, pointing out that although no mechanisms have been proposed, *ACSL1* gene polymorphism was strongly associated with an improvement in VO_2max_ after the 20-week exercise program [48]. Thus, this current work accedes to the Lefai et al. study’s conclusions that *ACSL1* might be one of the key cellular regulators involved in fitness enhancement and fat oxidation in response to aerobic exercise. Furthermore, our conclusions are expanded to include an enhancement of fat loss by the *ACSL1* gene variant, rs116143768, in untrained women with normal weight in response to aerobic exercise. In addition, the fat loss in our study is attributed to aerobic training alone because the diet was an isocaloric diet, meaning that our participants were not asked to lower their calorie intake but were obliged to perform aerobic training.

To our knowledge, the remaining genes with a suggestive *p*-value probably do not relate to lipid metabolism. According to the NCBI server [57], *PTPRZ1* encodes protein tyrosine phosphatase receptor type Z1. Its expression is restricted to the central nervous system (CNS), and it may be involved in the regulation of specific developmental processes in the CNS. *KANK1* encodes protein KN motif and ankyrin repeat domains 1, which functions in cytoskeleton formation by regulating actin polymerization. This gene is a candidate tumor suppressor for renal cell carcinoma. Mutations in this gene cause cerebral palsy spastic quadriplegic type 2, a central nervous system development disorder. *TENT5A* encodes terminal nucleotidyltransferase 5A, which enables RNA binding activity; is predicted to be involved in mRNA stabilization; is predicted to act upstream of or within the response to the bacterium; and is implicated in lung non-small cell carcinoma, osteoarthritis, and osteogenesis imperfecta type 18. *GALR* encodes galanin receptor 1 involved in a range of biological effects by interaction with specific G-protein-coupled receptors. Galanin receptors are seven-transmembrane proteins shown to activate a variety of intracellular second-messenger pathways. The last one, *LINC00478*, is broadly expressed in the brain and ovaries. None of the following variants *PTPRZ1* rs79300430, *KANK1* rs7867795, *TENT5A* rs112141659, *GALR1* rs144060810, or *LINC00478* rs238997 reached the genome-wide significant threshold (i.e., *p* < 5.0 × 10^−8^), likely due to no link with fat metabolism. 

Finally, a number of important limitations need to be considered. First, MAF in our study was of low frequency. This might impose a question of a more stringent statistical threshold for association testing since for populations with European ancestry, a significance threshold of 5 × 10^−8^ hits at ≥5% MAF [58]. Next, according to the Bayes theorem, the likelihood that an observed association actually exists in the sampled population depends not only on the reported *p*-value for the association but also on the sample size to detect the association [59]. Further work needs to include a replication study with a longitudinal design to establish whether a genotype–phenotype (*ACSL1* rs116143768-fat loss) observed association represents a credible association. This will require a higher number of participants. The current study only examined women with normal BMI, which represents a specific cohort. Ideally, they should be subjects with higher fat mass and BMI. Nonetheless, BMI alone does not reveal anything about the body’s measurements. Body composition is crucial for fat mass loss detection because lean body mass might be maintained (or even increased as in our study) while overall weight is lost [60].

## 5. Conclusions

In this investigation, the aim was to determine DNA polymorphisms associated with fat loss efficiency in untrained women with normal weight in response to a 12-week aerobic training program, followed by a cross-sectional study in athletes. The GWAS approach presented here identified that the T allele of *ACSL1* rs116143768 is associated with higher efficiency of fat loss in response to aerobic exercise in non-trained women and lower BMI in physically active men. Our results demonstrate a relationship between such a genotype and a predisposition to greater fat mass loss. Understanding the genetics of catabolic processes in the fat tissue would have a wide impact on the individualization of training programs to be more effective and safer, improving recovery, medical care, traumatology, nutrition, supplementation, and many other fields [61].

## Figures and Tables

**Table 1:** Changes in body composition in 126 women during a 12-week aerobic training period.

Parameters	Before Training	After Training	% Change from Baseline	*p*
Body mass, kg	60.94 (7.09)	60.33 (6.94)	−0.94 (2.44)	<0.0001 *
BMI, kg/m^2^	21.71 (2.44)	21.50 (2.39)	−0.92 (2.41)	<0.0001 *
FM, kg	15.13 (4.83)	14.28 (4.85)	−5.53 (11.77)	<0.0001 *
FM%	24.32 (5.03)	23.13 (5.09)	−4.68 (9.53)	<0.0001 *
FFM, kg	45.81 (2.95)	46.15 (3.03)	0.79 (2.99)	0.0044 *

Data are mean (SD). * *p* < 0.05, statistically significant changes after intervention.

**Table 2:** Summary statistics of the top SNPs associated with changes in body composition.

Gene/Near Gene	SNP	Chromo-some	Allele 1/ Allele 2	MAF	FM Change, %		FFM Change, %	
					beta	*p*	beta	*p*
*ACSL1*	rs116143768	4	T/C	0.0158	−27.55	1.18 × 10^−9^ *	6.91	2.35 × 10^−6^
*PTPRZ1*	rs79300430	7	G/T	0.0082	−34.91	6.14 × 10^−8^	7.32	4.19 × 10^−4^
*KANK1*	rs7867795	9	G/A	0.02	−21.81	1.48 × 10^−7^	6.13	3.41 × 10^−6^
*TENT5A*	rs112141659	6	G/A	0.0317	−17.01	3.11 × 10^−7^	3.25	2.59 × 10^−3^
*GALR1*	rs144060810	18	T/C	0.0119	−27.17	3.27 × 10^−7^	8.33	6.55 × 10^−7^
*LINC00478*	rs238997	21	T/C	0.0158	−22.96	7.46 × 10^−7^	5.36	3.26 × 10^−4^

* *p* < 5 × 10^−8^, genome-wide significant association; MAF, minor allele frequency; beta refers to the A1 allele.

## Data Availability

The data presented in this study are available on request from the corresponding authors.
